# Effects of Anesthesia on Cerebral Blood Flow and Functional Connectivity of Nonhuman Primates

**DOI:** 10.3390/vetsci9100516

**Published:** 2022-09-22

**Authors:** Xiaodong Zhang

**Affiliations:** EPC Imaging Center and Division of Neuropharmacology and Neurologic Diseases, Emory National Primate Research Center, Emory University, 954 Gatewood RD, Atlanta, GA 30329, USA; xzhang8@emory.edu; Tel.: +404-712-9874

**Keywords:** ketamine, isoflurane, alfaxalone, rsfMRI, monkey, functional connectivity

## Abstract

**Simple Summary:**

Nonhuman primates (NHPs) mimic most aspects of the human and play a critical role in translational research of neurological diseases. Functional connectivity is frequently examined using resting-state functional MRI (rsfMRI) to assess the functional abnormality in the anesthetized animal brain. As anesthetics show different effects on physiology and neural activity in a dose-dependent manner, specific and safe anesthesia procedures must be considered and applied to keep the animal from stress and motion during rsfMRI scanning. In this review, typical anesthesia protocols and rsfMRI techniques for NHPs are summarized. An optimal anesthesia protocol and rsfMRI scanning protocol for NHPs are introduced.

**Abstract:**

Nonhuman primates (NHPs) are the closest living relatives of humans and play a critical and unique role in neuroscience research and pharmaceutical development. General anesthesia is usually required in neuroimaging studies of NHPs to keep the animal from stress and motion. However, the adverse effects of anesthesia on cerebral physiology and neural activity are pronounced and can compromise the data collection and interpretation. Functional connectivity is frequently examined using resting-state functional MRI (rsfMRI) to assess the functional abnormality in the animal brain under anesthesia. The fMRI signal can be dramatically suppressed by most anesthetics in a dose-dependent manner. In addition, rsfMRI studies may be further compromised by inter-subject variations when the sample size is small (as seen in most neuroscience studies of NHPs). Therefore, proper use of anesthesia is strongly demanded to ensure steady and consistent physiology maintained during rsfMRI data collection of each subject. The aim of this review is to summarize typical anesthesia used in rsfMRI scans of NHPs and the effects of anesthetics on cerebral physiology and functional connectivity. Moreover, the protocols with optimal rsfMRI data acquisition and anesthesia procedures for functional connectivity study of macaque monkeys are introduced.

## 1. Introduction

Resting-state functional MRI (rsfMRI) can examine the intrinsic synchronous activations between regions that are spatially distinct in the brain and has become a robust tool to assess the functional organization and status of the central nervous system in preclinical and patient studies. In particular, with the advancement of contemporary neuroimaging techniques with high and ultrahigh-field MRI, it is increasingly used to examine brain function and disorders in small and large animal models, including mice, rats, pigs, and nonhuman primates (NHPs). However, animals are usually scanned under general anesthesia to keep them from stress and motion and minimize motion artifacts [1]. Due to the substantial difference in the characteristics and biological activity of each anesthetic agent, specific anesthesia protocols have been established for each species [2,3,4,5].

General anesthesia is used in both humans and animals to induce unconsciousness and keep the subject from feeling pain or stress during medical procedures. In functional MRI studies, the human is usually scanned awake for a short period. In contrast, animals are generally anesthetized for structural and functional MRI study using total intravenous anesthesia (TIVA) or inhalational anesthesia to keep them from stress and moving during scanning for up to several hours. Extensive studies have demonstrated that cerebral blood flow (CBF), metabolism, and neuronal function are substantially affected by anesthesia [6,7,8]. In particular, brain neural activity can be suppressed by most anesthetics [9,10,11,12,13]. Species differences in response to anesthetics have been observed between humans and rodents in a previous fMRI study of mice [14]. Functional connectivity showed strong dependence on the type and depth of anesthesia, as reported in previous studies of rats [15] and mice [12]. Therefore, proper anesthesia is critical and highly demanded for optimal detection of rsfMRI signals in animal models during data collection.

NHPs are our closest living relatives and resemble most aspects of humans in neuroanatomy, physiology, immunology, metabolism, etc. Rhesus macaques share 93.5% of the human genome [16]. NHP models show a superior and unique advantage in translational research compared to rodents and other large animals and are widely used in neuroscience research and pharmaceutical development [17,18,19,20]. NHPs models have been used for studies in immunology and vaccine development [18,21], neuroAIDS [22,23,24,25], stroke [26,27,28], autism [29,30], Huntington’s disease [31,32], Alzheimer disease [33,34,35], and Parkinsonian disease [36,37].

Altered functional connectivity of the default mode network (DMN) is seen in the brain with neurological disorders and correlated with cognitive status [38,39]. DMN includes the medial prefrontal cortex (mPFC), posterior cingulate cortex (PCC)/precuneus, ventral anterior cingulate cortex (ACC), medial, lateral, and inferior parietal cortex [40]. Monkeys showed a functional equivalent of the human DMN [41]. The intrinsic functional connectivity within three well-known systems (oculomotor, somatomotor and visual) and the default mode networks of the human brain were also observed in anesthetized rhesus monkeys [11]. Many studies of NHPs demonstrated that rsfMRI has a unique capacity to assess the functional alteration of anesthetized NHP models with neurological dysfunction [42,43,44].

The aim of this review is to summarize anesthesia protocols used in functional connectivity studies of NHPs with rsfMRI and the effects of anesthetics on cerebral physiology and functional connectivity. As the neural activity in the brain is closely associated with cerebral physiology, the information about the effects of anesthesia on physiology and rsfMRI signal could be helpful in rsfMRI experimental design and data interpretation of neuroscience studies using NHP models.

## 2. Methodology

A literature search on the NIH national library of medicine website (www.pubmed.gov, accessed on 1 September 2022) was conducted with keywords including ‘nonhuman primate (or nonhuman primate), functional connectivity, resting state’. Articles published from 2005 to 2022 (31 August 2022) were considered, and 137 results were found. These articles were manually sorted through. In addition, the site of Web of Science (www.webofscience.com, accessed on 12 September 2022) was searched using the same keywords and periods, and 6 additional publications were identified manually and included. Fifty articles were confirmed and selected in this review.

### 2.1. MRI Techniques to Examine Physiology and Functional Connectivity of Anesthetized NHP Brains

Progress in modern MRI techniques in hardware and software advances neuroimaging studies of animals and humans substantially and benefits the rsfMRI data acquisition and application [45]. Squirrel monkeys and marmosets can be scanned with animal scanners such as 9.4T to exploit the advantage of ultrahigh-field (7T or higher) fMRI techniques. Macaque monkeys are mostly conducted in clinical 3T settings in which parallel imaging techniques are generally implemented. Multiband EPI sequences can be used to increase the sampling rate to improve fMRI signal detection [46]. Multi-echo EPI allows for utilizing the TE-domain information to enhance fMRI analysis compared to traditional EPI in rsfMRI studies [47]. Cerebral physiology, including CBF, cerebral blood volume, CMRO2, and oxygen extraction fraction, can be measured using perfusion MRI, BOLD fMRI, and cerebral oxygen extraction fraction MRI techniques in humans and animals [48,49]. Functional connectivity can be estimated quantitatively using rsfMRI, positron emission tomography (PET), near-infrared spectroscopy (NIRS), electroencephalography (EEG), and magnetoencephalography (MEG) [50]. Among these techniques, rsfMRI is a noninvasive approach with high spatial resolution, widely used in neuroscience research with humans and animals [51,52].

### 2.2. Resting-State Functional MRI

Resting-state functional MRI (rsfMRI) can detect the spontaneous fluctuation of the blood oxygenation level-dependent (BOLD) signal in the brain and provide an indirect measure of brain activity [53]. Functional connectivity demonstrates the statistical dependence of the rsfMRI signals between different brain regions and has been used widely to assess brain function alterations in various neuroscience studies of animals and humans [54,55,56]. In addition, rsfMRI is often exploited to examine the functional connectivity in anesthetized rodents on ultrahigh-field animal MRI scanners [13,55,57] and monkeys of either healthy [58,59] or disease models [42,43] using clinical 3T MRI settings.

It is well known that anesthesia usually suppresses the neural activity of the brain in a dose-dependent manner compared to the awake state of the brain [10,11,12]. In order to improve the rsfMRI signal detection in anesthetized animals, different anesthesia procedures have been explored to minimize the suppression effects. Dexmedetomidine and medetomidine (used alone or combined with isoflurane) are suggested to be optimal choices for rsfMRI in rodent models [57,60]. Furthermore, as the BOLD signal changes are proportional to the magnetic field strength [61], ultrahigh-field (7T and above) MRI is recommended to improve the detection sensitivity of rsfMRI for anesthetized brains.

**Figure 1:**
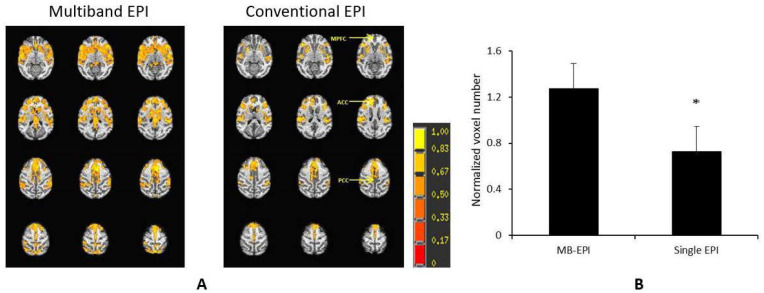
Demonstration of the multiband MRI technique to improve detection sensitivity of BOLD signal in anesthetized monkeys on a Siemens TIM Trio 3T scanner with an 8-channel array volume coil. (**A**) Normalized correlation maps of the default mode network (DMN) of anesthetized (1% isoflurane) rhesus monkeys scanned by using the multiband echo planar imaging (multiband EPI) sequence with the parameters: TR = 1059 ms/TE = 25 ms, MB factor = 4, voxel size=1.5 mm × 1.5 mm × 1.5 mm, time of acquisition = 10 min), and conventional single-shot EPI acquisition (conventional EPI) with TR = 2190 ms/TE=25 ms, voxel size=1.5 mm × 1.5 mm × 1.5 mm, time of acquisition = 10 min. The z-score threshold: *p* < 8 × 10^−18^ plus cluster threshold 169 mm^3^/overall, mean ± stdev. *n* = 4. *: *p* = 0.08, paired *t*-test. (**B**) Comparison of normalized voxel numbers (normalized by average voxel numbers across DMN from the two EPI acquisitions (multiband EPI vs. single EPI). MB, multiband.

However, ultrahigh-field MRI scanners are mostly built with small-bore sizes for small animals (such as rodents) and are not applicable to large animals in most institutes. In contrast, clinical 3T high-field MRI scanners are installed widely in imaging research facilities or clinics. They are mostly used for scanning macaque monkeys, one of the mostly used primate species in neuroscience and biomedical research. Previous studies have demonstrated that the multiband MRI technique can substantially improve the rsfMRI detection sensitivity and statistical power in studies of the human connectome. One previous study indicated that the multiband MRI technique could also improve the sensitivity for rsfMRI examination in anesthetized macaque monkeys using a clinical 3T setting (Figure 1) [62].

### 2.3. Perfusion MRI

CBF quantifies the blood supply to the brain and is closely associated with neural activity [8,9,63,64]. CBF can be measured using arterial spin-labeling (ASL) or dynamic susceptibility contrast-enhanced (DSC) perfusion MRI techniques [65]. Unlike DSC-MRI, ASL-based perfusion MRI measures CBF by using RF-tagged blood water in the artery as an endogenous contrast agent. Therefore it allows for examining the dynamic changes of CBF with high spatial and temporal resolution [63,66] with quantification [67].

ASL perfusion can be conducted for monkeys using a three-coil or two-coil setting in which a separate labeling coil is applied to tag the blood water using a clinical 3T scanner [68,69]. Moreover, ASL perfusion MRI can be implemented using the pseudo-continuous ASL MRI technique (pCASL) including the 3D gradient and spin-echo (3D-GRASE) ASL acquisition on clinical scanners in which the additional RF-labeling coil is not required [70], offering the convenience for perfusion data collection of monkeys on a conventional 3T scanner.

## 3. Effects of Anesthesia on CBF and Functional Connectivity of NHPs

Inhalational anesthesia, including nitrous oxide, halothane, isoflurane, sevoflurane, desflurane, and intravenous/intramuscular anesthesia, including ketamine, alphaxolone-alphadolone, propofol, barbiturate, and alfolaxone, have been used for diagnosis purpose or biomedical research of NHPs. To date, isoflurane, ketamine, and propofol are among the most used anesthetics for general anesthesia of laboratory animals [71] and neuroimaging studies of NHPs as well [5,72]. They may be administrated intravenously (IV) or through a mask/tube for several hours in a single scanning session for data collection with multiple modalities, including high-resolution structural MRI, diffusion and perfusion MRI, in vivo MR spectroscopy (MRS), and rsfMRI. The protocols for isoflurane, propofol, and ketamine administration are well established for NHPs [5,72,73]. Meanwhile, anesthetics likely induce unconsciousness at both the subcortical and cortical levels, acting on subcortical areas to decrease arousal while simultaneously affecting the cortex to degrade the contents of consciousness [74].

Compared to structural MRI, rsfMRI of animals often requires stable control of physiology and accurate maintenance of depth of anesthesia during data collection. As seen in the selected 50 literatures for functional connectivity studies using NHPs (macaque monkeys, squirrel monkeys, marmosets, vervet monkeys, capuchin monkeys, baboons, and chimpanzees), six studies of NHPs, such as marmosets [75,76] and rhesus monkeys [77,78], were conducted awake. All others were conducted under anesthesia. Isoflurane was mostly utilized in rsfMRI studies of NHPs (39 of 50 studies), including squirrel monkeys [79], marmosets [80], vervet monkeys [81], neonatal and infant rhesus monkeys [44,82], adult rhesus monkeys [43], baboons, capuchin monkeys, and chimpanzees [83]. In contrast, very few used intravenous anesthetics in the rsfMRI data collection of NHPs. As seen in the selected literatures, three rsfMRI studies used propofol (IV, 0.25 mg/kg/min) for adult rhesus monkeys [84] and marmosets [85], and one study used medetomidine (10 μg/kg) and ketamine (10 mg/kg) for rsfMRI and diffusion MRI scans of adult rhesus monkeys [86]. In addition, one study used ketamine (10 mg/kg, IM) and Zoletil 50 tiletamine-zolazepam (0.2 mg/kg) (for maintenance) in a previous rsfMRI study of adult spider monkeys at 3T [87].

Previous human studies have demonstrated the effects of isoflurane and propofol on CBF and the metabolism of the brain by using PET [88,89] or perfusion MRI [90,91] and MR spectroscopy techniques [92]. Significant and different effects of anesthetics (including volatile anesthetics such as isoflurane, sevoflurane, and desflurane, and intravenous anesthetics such as ketamine, propofol, Alfaxalone, and dexmedetomidine) on physiology and neuronal function are observed in animals and humans [7]. Currently, using ketamine as an induction agent followed by light isoflurane anesthesia is a typical anesthesia procedure used in fMRI and rsfMRI studies of macaque monkeys [11,93]. The procedure was also used to assess the functional brain abnormality of macaque monkey models in a clinical 3T setting [42,43] and functional connectivity studies of squirrel monkeys at 9.4T [79,94].

### 3.1. Isoflurane

Isoflurane is a volatile inhalation anesthetic agent commonly used in medical procedures and in vivo neuroimaging examinations of experimental animals for its low blood gas solubility (i.e., rapid induction of and recovery from anesthesia), precise control of anesthetic depth, and minimal hepatic metabolism [95]. Induction of isoflurane can result in evident effects on cerebral blood flow (CBF), cerebral blood volume (CBV), permeability, neurovascular coupling, and neuron functionality [96,97,98,99,100,101,102]. Increased CBF and reduced rate of cerebral oxygen consumption (CMRO2) have been observed in animals and humans anesthetized with isoflurane [100,102]. A disturbed CBF autoregulation mechanism was seen in previous studies of canines, baboons, and humans under high doses of isoflurane [102,103,104]. In addition, isoflurane shows greater sensitivity to evoked cortico-cortical responses compared to thalamo-cortical responses [105].

The 0.75–1.5% isoflurane, mixed with 100% O_2_ or ambient air, is normally used as the maintenance dose for sedation purposes during MRI scanning. A prior Xenon-133 study of anesthetized baboons has shown that isoflurane produces vasoconstriction in cerebral vessels in low doses (~0.5% or less) and vasodilation in high doses (~0.95% or higher) [104], indicating isoflurane has a biphasic characteristic. Accordingly, the vasodilation effect is dominant when ~1% or higher doses are applied. Reinstrup et al. observed that relative CBF was increased with 1.0 MAC isoflurane in subcortical regions of humans by using SPECT imaging [106,107]. Li et al. further investigated the dose-dependent effect of isoflurane on regional CBF in monkeys using ASL perfusion MRI [69]. They found CBF in the thalamus and cerebellum increased by about 39% and 55% (0.75% vs. 1.5% isoflurane). However, there was no observable CBF change in cortical regions, suggesting subcortical structures are more susceptible to isoflurane. When the isoflurane dose is further increased to 2%, a significant CBF increase is also seen in cortical regions [70], indicating that autoregulation in cortical regions can be disrupted when a higher dose of isoflurane is applied. Isoflurane showed dose-dependent suppression effects on human and primate brains’ neural activity and functional connectivity [10,11,108,109].

In addition, monkeys may be anesthetized for several hours for neuroimaging data collection, as seen in prior studies that reported the exposure of neonatal monkeys to sevoflurane for 5 h [110] and to isoflurane for up to 7 h in a stroke study with rhesus monkeys [111]. Li et al. reported that prolonged administration of isoflurane could alter the CBF distribution in the cortical and subcortical regions and attenuate the rsfMRI signal of anesthetized monkeys [112], suggesting data collection for rsfMRI should be conducted early for better sensitivity; the timing with CBF and rsfMRI acquisition should be kept consistent if administration of anesthesia is substantially prolonged. The possible reasons for the effects of long-duration administration of isoflurane may be because the anesthetic potency of isoflurane is highly correlated with the lipid bilayer partition. As a longer duration of anesthesia allows for a higher concentration of isoflurane in the lipid tissue of the nervous system, neural activity might decrease progressively over the duration. Accordingly, CBF is reduced because of its close coupling with the brain metabolism in a normally functioning brain. These CBF reduction effects should be considered when monkeys are under prolonged scans.

Sevoflurane and desflurane also provide good control of anesthetic depth and rapid recovery from anesthesia [113] and are commonly used in clinical practices and preclinical studies [114,115,116]. Sevoflurane, desflurane, and isoflurane showed different cerebral vasodilation effects [117]. Compared to isoflurane, sevoflurane does not have a pungent smell and is not irritating to the respiratory tract. Therefore, sevoflurane can be an alternative for mask induction with an induction chamber and maintenance of New World primates (such as marmosets) [118]. Previous PET studies of adult rhesus monkeys suggested sevoflurane exposure has minimal effect on cognitive function [119], and the suppression effects of sevoflurane on the BOLD signal and intrinsic neural activity of the human brain were seen [120]. Therefore, it is warranted to be explored for more NHP models of neuroscience research in the future. Meanwhile, desflurane enables the most rapid onset of anesthesia and recovery due to its lowest solubility in blood. It shows an advantage for anesthesia in elderly patients with long-duration operations [121] and is less neurotoxic than isoflurane to developing brains [122]. It is also suitable for neurosurgery of rhesus monkeys [123]. However, desflurane is highly volatile and requires special vaporizers for administration [124], and it is also more pungent and irritating to the airway tract compared to isoflurane. Therefore, desflurane is rarely used in neuroimaging studies of NHPs. However, desflurane may be an alternative for anesthesia in some NHP models for its special property, and its effects on functional connectivity will be investigated accordingly in the future.

### 3.2. Ketamine

As an NMDA receptor agonist in the thalamoneocortical and limbic systems, ketamine is a fast-acting dissociative anesthetic used as an induction and maintenance agent in veterinary anesthesia, with neuroprotective and antidepressant effects [125]. Ketamine is primarily eliminated by the kidney with a mean terminal half-life of ~155 min in humans (in a dose of 0.5 mg/kg) [126]. It is often combined with xylazine as an injectable anesthetic for the anesthesia of rodents [127]. In addition, ketamine shows a direct vasodilatory effect on the cerebral vasculature and global and regional CBF increase is generally seen in animals and humans [128,129]. A recent rsfMRI study showed that ketamine decreases the connectivity of default mode network in the human brain [130]. Ketamine’s effects on some function domains have been investigated in humans and animals as it showed the potential for treatment in depressive disorders when administrated with sub-anesthetic doses [131].

It is a typical procedure to use ketamine as an induction agent prior to inhalation anesthesia in large animals such as NHPs. Additionally, ketamine showed a vasodilation effect and increased CBF in large animals such as spontaneously-breathing goats [132] and rabbits (1 mg/kg, IV) [133]. Li et al. reported significant CBF reduction in most cortical and subcortical regions of rhesus monkey brains when the anesthetic was switched from ketamine (1.6 mg/kg/min, IV) to isoflurane (~0.8%) [134], indicating ketamine shows a stronger vasodilation effect than isoflurane.

Furthermore, ketamine’s suppression effect on functional connectivity of the default mode network was seen in humans (S-ketamine, 0.25 mg/kg, IV) [135], and the BOLD signal change was in a dose-dependent manner (racemic ketamine hydrochloride solution (1 mg/mL)) [136]. A human fMRI/EEG study reported decreased functional connectivity in the medial prefrontal cortex (mPFC), whereas increased connectivity was observed in intraparietal cortices after the administration of subanesthetic S-ketamine [130]. A rhesus monkey study reported ketamine increased functional connectivity between the dorsolateral prefrontal cortex (dlPFC) and several cortical and subcortical regions during sub-anesthetic IV infusion of ketamine (0.345 mg/kg bolus followed by 0.256 mg/kg/h constant infusion) [137]. Moreover, a significant increase in prefrontal-hippocampal connectivity was seen in a rsfMRI study of humans and rats administrated with ketamine (0.5 mg/kg for humans, 25 mg/kg for rats, IV) [138]. The previous findings suggest that the effects of ketamine on functional connectivity are region- and dose-dependent.

Li et al. also investigated the effect of ketamine on the functional connectivity of rhesus monkeys when ketamine was used alone as an induction and maintenance agent and later switched to isoflurane [134]. Interestingly, no significant changes in functional connectivity in the default mode network (MPFC-PCC, ACC-PCC, and PCC-PCC) were observed when switching the maintenance anesthetic from ketamine (1.6 mg/kg/min, IV) to isoflurane (0.8%) for the 4 rhesus monkeys.

Ketamine can be combined with medetomidine during the anesthesia of macaque monkeys as medetomidine provides muscle relaxation but not causing cardiovascular and respiratory effects [139]. In addition, dexmedetomidine has been used widely as a sedative agent in fMRI and functional connectivity studies of rodents [60,140] by combining it with isoflurane and/or ketamine. These combinations of procedures were explored in a few studies of monkeys for fMRI [141,142] and functional connectivity [86], suggesting it may improve the detection sensitivity of BOLD signal in the monkey brain at 3T. In addition, ketamine is a racemic mixture of R- and S- ketamine and both showed suppression effects on fMRI signals in the brain. Previous studies of animals and humans showed that racemic ketamine and S-ketamine increased CBF in the brain, and cerebrovascular effects of racemic ketamine can be complicated by the background anesthetics [143,144]. Interestingly, different fMRI responses of two enantiomers were observed in conscious rats [145], suggesting the FC in the brain may be affected differently by them, and the difference should be considered in rsfMRI studies of NHPs. Additionally, racemic ketamine altered functional connectivity similarly as seen in psychotic patients [146].

### 3.3. Propofol

Propofol is a popular anesthetic used in animals, including NHPs. It was applied previously for general anesthesia of NHPs in long structural and diffusion MRI scans of stroke macaque monkeys [147] and a study of resting-state brain activity in chimpanzees for PET and MRI scans [148]. Previous human studies suggested it results in reduced CBF and functional connectivity in the default mode network and salience network but increased connectivity in the motor and visual network [149]. A previous rat study demonstrated that propofol caused multiphasic, dose-dependent changes in functional connectivity of various cortical and subcortical networks [150]. Compared to isoflurane, propofol is not widely used as a maintenance agent in the neuroimaging study of NHPs. As seen in the selected literature of NHPs, propofol was just used as a maintenance agent in a few previous studies of rhesus monkeys which received ketamine (IM, 10 mg/kg) for induction and maintained under propofol (IV, 0.25 mg/kg/min) for FC-MRI scan [84].

### 3.4. Alfaxalone

Alfaxalone (also known as alphaxalone or alphaxolone) is a synthetic neuroactive steroid anesthetic, and Alfaxalone-2-hydroxpropyl-β-cyclodextrin (Alfaxalone-HPCD) is its newest formulation and was approved by the FDA for use in dogs and cats in 2012 [151]. Alfaxalone produces satisfactory induction and maintenance of anesthesia, such as the onset of anesthesia, fast redistribution, short elimination half-life, and short duration of action [152], and has been explored for anesthesia of horses [153], dogs [154], pigs [155], cats [152,156], and NHPs [157,158,159], suggesting alfaxalone has the potential to become an alternative anesthetic for induction and maintenance anesthesia of large animals. The pharmacological and anesthetic effects of alfaxalone in dogs were reviewed recently [160].

Li et al. investigated the effects of alfaxalone on CBF and intrinsic neural activity in rhesus monkeys and compared it to ketamine [134]. Dramatic CBF changes in cortical and subcortical regions during the administration of alfaxalone (or ketamine) and successive isoflurane exposure were observed. Obviously, CBF was affected by alfaxalone very differently from ketamine. The mean CBF during alfaxalone administration was substantially lower than that with ketamine in the cortical and subcortical regions. Additionally, the mean CBF substantially increased in grey matter and white matter after alfaxalone was replaced by isoflurane exposure in the same animals, indicating alfaxalone has controversial effects on CBF compared to ketamine as an induction agent for NHP anesthesia. Interestingly, the previous rsfMRI study of rhesus monkeys (*n* = 4) showed no significant difference in functional connectivity was observed in the interested default mode networks of the brain anesthetized by comparing alfaxalone to ketamine (~1.6 mg/kg/min, IV) and isoflurane (~0.8%) [134], probably due to the small sample size (*n* = 4).

Compared to ketamine, alfaxalone showed an evident vasoconstriction effect, as reported in a pig study [161]. The combined use of alfaxalone and alfadolone decreased CBF substantially in cats measured by 133Xe [162]. Moreover, a recent report showed that alfaxalone resulted in significantly lower CBF in dogs in comparison with isoflurane [163]. Neural activity in the brain is usually coupled with local CBF increase, the basis of a BOLD signal generated in the vasculature [164]. As ketamine shows stronger vasodilatory effects than isoflurane and could affect the fMRI results of animals under hypercapnia, alfaxalone may be an alternative induction agent for the affected NHP models and should be further explored in the future. In addition, the common marmoset, a small New World monkey, is increasingly used in neuroscience and can be scanned awake or anesthetized in animal MRI scanners in which ultrahigh field MRI techniques can be exploited to investigate the effects of anesthesia on CBF and neural activity in the brain [80,94,165].

Previous studies have reported that anesthesia exposure in young children (<3 or 4 years old) is an important factor in developmental deficits such as learning disabilities [166] and an increased incidence of attention deficit hyperactivity disorder (ADHD) [167]. Long-term adverse effects of anesthesia on cognitive behavior might be more related to repeated exposure in neonatal subjects [168,169]. Therefore, the administration duration of anesthesia should be optimized to minimize its adverse effects on developing brains when scanning postnatal and young primates.

Due to the substantial suppression effects of isoflurane on neural activity, a better anesthesia protocol is highly demanded to improve the BOLD signal detection sensitivity. Medetomidine and dexmedetomidine are alpha-2 adrenoreceptor agonists and showed improved sensitivity to detect BOLD responses [60,170]. Dexmedetomidine was also explored in a previous fMRI study of macaque monkeys at 3T by a combination of isoflurane (0.25%) with dexmedetomidine (15 μg/kg/h, IV) [141] and the results demonstrated it is promising in improving the detection of BOLD signal in anesthetized NHPs, suggesting further investigation for their use in routine rsfMRI examination in NHPs is warranted in future studies.

## 4. Conclusions

NHPs are among the most challenging species and play a key and unique role in neuroscience research [17]. As NHPs are costly and rare resources, careful justification of the sample size for their usage is always required, and the sample size can be limited to as few as 3–4 animals for a research project. Resting-state functional MRI provides a noninvasive and robust approach to examining functional abnormality in the NHP brain. Although ultrahigh-field MRI scanners can improve the detection sensitivity of fMRI signals, they are still not available for most NHP neuroimaging studies nowadays. Therefore, an fMRI protocol with proper anesthesia procedure and optimized EPI pulse sequence is essential to ensure successful rsfMRI data collection of anesthetized NHPs in a conventional 3T clinical setting.

Compared to rodents, the option of anesthesia protocols for the rsfMRI study of NHPs is limited. The protocol with ketamine (IM) and isoflurane is a well-established and popular procedure and has been used for most rsfMRI studies of anesthetized NHPs, including infant and adult macaque monkeys, squirrel monkeys, and marmosets which are the primate species mostly used in neuroscience research. It is worth mentioning that this anesthesia protocol is generally being used in routine structural and fMRI scans of macaque monkeys aged from one week to over 20 years old in the author’s institute. However, due to the strong vasodilation effect of ketamine, it may be adverse and not applicable in some models of NHPs and alfaxalone could be an alternative to induction agent prior to isoflurane administration accordingly.

## Data Availability

The data presented in this study are available on request from the corresponding author.
